# Synergistic Effect of Metronidazole and Chlorhexidine against *Porphyromonas gingivalis* Growth: An In Vitro Study

**DOI:** 10.3390/dj12100307

**Published:** 2024-09-27

**Authors:** Claudia Lorenzi, Fabrizio Lio, Vincenzo Mazzetti, Paolo Carosi, Stefano Lamelza, Enrico Salvatore Pistoia, Francesca Pica, Roberta Gaziano

**Affiliations:** 1 Department of Chemical Science and Technologies, University of Rome Tor Vergata, 00133 Rome, Italy; claudia.lorenzi@alumni.uniroma2.eu (C.L.); fabrizio.lio@alumni.uniroma2.eu (F.L.); vincenzo.mazzetti@alumni.uniroma2.eu (V.M.); 2 Department of Clinical Sciences and Translational Medicine, School of Dentistry, University of Rome Tor Vergata, 00133 Rome, Italy; 3 Private Practice, Colleferro, 00034 Rome, Italy; stefano-sw@hotmail.it; 4 Department of Experimental Medicine, University of Rome Tor Vergata, 00133 Rome, Italy; pistoiae@uniroma2.it (E.S.P.); pica@uniroma2.it (F.P.); gznrrt00@uniroma2.it (R.G.)

**Keywords:** *Porphyromonas gingivalis*, periodontitis, chlorhexidine, metronidazole, synergistic effect

## Abstract

**Aim:** To evaluate the potential synergistic activity of metronidazole (MTZ) and chlorhexidine (CHX) against *Porphyromonas. gingivalis (P. gingivalis)* growth. **Methods:** Antimicrobial susceptibility tests of *P. gingivalis* to MTZ and CHX were performed on in vitro serial 2-fold dilutions of MTZ (from 1 mg/mL to 0.015 mg/mL) and CHX (from 1 mg/mL to 0.03 mg/mL) in thioglycollate medium broth in a 96-well plate. The turbidity of each sample was analyzed by absorbance spectrophotometry at 450 nm wavelengths by using an enzyme-linked immunosorbent assay (ELISA) reader. The MIC50 (minimum inhibitory concentration) and MBC (minimum bactericidal concentration) were assessed. To investigate the potential synergism between MTZ and CHX, bacterial cells were treated with MTZ or CHX, as described above, either alone or in combination. **Results:** The MIC_50_ of MTZ was 0.03 mg/mL while that of CHX ranged from 0.12 to 0.06 mg/mL. MTZ and CHX exerted a significant inhibitory effect on *P. gingivalis* growth in a dose-dependent manner. MTZ at a low and ineffective concentration of 0.015 mg/mL, associated with a suboptimal concentration of CHX (0.03 mg/mL), exhibited a significant synergistic inhibitory effect on bacterial growth (50% inhibition vs. control) (*p* < 0.001), and the effect was more remarkable with 0.06 mg/mL CHX (75% inhibition vs. control). **Conclusions:** CHX and MTZ showed a significant synergistic effect against *P. gingivalis* growth. A non-effective concentration of MTZ (0.015 mg/mL) combined with suboptimal concentrations of CHX (0.03 mg/mL and 0.06 mg/mL) were related to a 50% growth in the inhibition and 99.99% death of *P. gingivalis*, respectively. The applicability of the clinical use of these concentrations should be tested in randomized controlled trials.

## 1. Introduction

Periodontal diseases are multifactorial oral pathological conditions characterized by the progressive resorption of tooth-supporting structures [1]. Periodontitis is a complex, multi-cause infection involving multiple microorganisms, marked by inflammation that destroys the tissues supporting the teeth. This condition leads to the formation of periodontal pockets, the loss of alveolar bone and, ultimately, to tooth loss [2]. It is also related to various systemic diseases, like diabetes mellitus and atherosclerosis [3,4]. Its role in tooth-supporting tissue destruction is associated with the action of subgingival dental plaque, which constitutes its most important etiological factor. *P. gingivalis* is a Gram-negative, anaerobic bacterium found in the mouth, and recognized as a major factor in the onset and advancement of periodontitis. It is responsible for many cases of chronic periodontitis [5,6]. A model of periodontitis pathogenesis based on polymicrobial synergy and dysbiosis has been proposed. Dysbiosis refers to an imbalance in the microbial community of the oral cavity. In a healthy state, there is a diverse and balanced microbiome. When dysbiosis occurs, the harmful bacteria outnumber the beneficial ones, leading to inflammation and tissue damage. Dysbiosis in periodontal disease is characterized by an overgrowth of pathogenic bacteria, which disrupts the homeostasis of the microbial community. This imbalance promotes chronic inflammation and contributes to periodontal tissue destruction. Factors, such as poor oral hygiene, smoking, and systemic conditions, can contribute to this dysbiosis. The founding hypothesis is that the disease starts from an imbalance between the host and oral microbiota, in which key species, such as *P. gingivalis*, *T. forsythia*, and *T. denticola*, cooperate in driving the selection of an inflammatory microbiota and, as a consequence, periodontal inflammation [7].

In vitro and in vivo studies have demonstrated how *P. gingivalis* can destroy periodontal tissue, escape the host’s innate and adaptive immunity, and cause alveolar bone resorption. *P. gingivalis* has a unique ability to modulate the immune system, triggering periodontal disease, and, thereby, promoting oral dysbiosis [8,9]. Indeed, colonization by *P. gingivalis* can disturb the normal balance of oral microbial communities, resulting in inflammation and bone loss [10].

Nonsurgical traditional treatment methods, including subgingival instrumentation (SRP), are not able to guarantee disease eradication [11]. Therefore, during STEP II of treatment, several antimicrobial agents with some potential to improve host defense immune responses have been investigated [12]. The systemic administration of antibiotics for the treatment of periodontitis is limited by the need to use high doses to achieve an appropriate drug concentration in the gingival fluid, the rapid increase in drug resistance among bacteria, and the side effects of the drugs [13]. Chlorhexidine (CHX) is a cationic bis-guanide antimicrobial agent with a broad spectrum of antimicrobial activity, showing inhibitory effects on dental biofilm formation [14]. Furthermore, CHX exerts proteolytic activity against certain periodontal pathogens and also exhibits an antioxidant capacity. A recent meta-analysis review showed that rinsing with 0.12% CHX after nonsurgical periodontal therapy reduces the probing depth (PPD) to a greater extent than nonsurgical periodontal therapy alone [15]. However, a recent experimental study by Coelho et al. demonstrated that chlorhexidine (CHX) can have cytotoxic effects on human gingival fibroblasts (HGF) at concentrations lower than those typically used in clinical practice [16]. Additionally, a CHX allergy can lead to type IV hypersensitivity reactions, such as a burning sensation in the mouth, erythema of the gingival tissues, and stomatitis. These reactions present challenges for both clinicians and patients [15].

Additionally, the use of systemic antibiotic therapies such as MTZ have shown clinical benefits in the treatment of periodontitis, leading to a significant reduction in periodontal pathogen levels compared to SRP alone. However, the systemic administration of MTZ may induce adverse effects in patients, such as headache, nausea, loss of appetite, diarrhea, or metallic taste [17].

The aim of this study was to evaluate the potential synergistic activity of CHX and MTZ against *P. gingivalis* growth in vitro for the development of novel antimicrobial approaches to the prevention/treatment of periodontitis that may reduce the adverse effects of these drugs.

## 2. Materials and Methods

### 2.1. Bacterial Strain and Culture Conditions

*Porphyromonas gingivalis* (ATCC 33277) cells maintained as frozen stock were firstly grown in thioglycollate medium broth (Thermo Scientific, Ann Arbor, MI, USA) in an anaerobic atmosphere composed of 10% H_2_, 5% CO_2_, and 85% N_2_ at 37 °C. After a 5-day culture, 50 mL of the bacterial culture broth was plated on Scheadler KV agar additioned with lysed horse blood (Thermo Scientific) and incubated for 4 days at 37 °C. Then, a single colony was picked from the thioglycollate medium broth and cultured in the same anaerobic conditions for 24–48 h.

### 2.2. Antimicrobial Susceptibility Test

For antimicrobial susceptibility, the bacterial cell suspension was adjusted to 0.5 McF (1.5 × 10^6^ cells/mL). Antimicrobial susceptibility tests of *P. gingivalis* to MTZ (Merck KGaA, Darmstadt, Germany) and CHX (Merck KGaA, Darmstadt, Germany) were performed according to the CLSI guidelines (Clinical and Laboratory Standards Institute, 2016). Briefly, serial 2-fold dilutions of MTZ (from 1 mg/mL to 0.015 mg/mL) and CHX (from 1 mg/mL to 0.03 mg/mL) were made in thioglycollate medium broth in a 96-well plate (Thermo Fisher Scientific, Waltham, MA, USA) with 50 mL of each compound. To assess the possible synergistic effect of MTZ and CHX against *P. gingivalis* in vitro, bacterial cells were treated with MTZ at doses ranging from 1 mg/mL to 0.015 or CHX from 1 mg/mL to 0.06 mg/mL, either alone or in combination. A volume of 100 mL of the bacterial suspension was added to each well. The untreated bacterial suspension (100 mL) was used as the reference control. Negative controls (100 μL of culture medium alone or 100 μL of culture medium plus 50 μL of each compound in the absence of bacterium) were included in all the experiments. Propylene glycol 75%/purified water 25%, sodium hyaluronate 0.5% plus purified water, and a water glycol solution 7525QB as the vehicles were used as the negative controls. The desired final volume was adjusted to 200 mL for each well. The microplate was incubated anaerobically at 37 °C for 48 h. After that, the turbidity of each sample was analyzed by absorbance spectrophotometry at 450 nm wavelengths by using an enzyme-linked immunosorbent assay (ELISA) reader (ThunderBolt, Tecan, Milan, Italy). Three independent experiments were carried out in triplicate. The MIC_50_ (minimum inhibitory concentration) was defined as the lowest concentration of the compounds capable of inhibiting 50% of the bacterial growth, compared to the drug-free control. The MBC (minimum bactericidal concentration) was defined as the lowest concentration resulting in the death of 99.9% or more of the initial inoculum. The MBC was determined by subculturing 50 μL from each well onto Schaedler agar plates. The plates were incubated in an anaerobic chamber at 37 °C. After 24 h, the cell viability was evaluated.

### 2.3. Statistical Analysis

A statistically descriptive analysis was performed. More specifically, the data were presented as the means ± SD of three independent experiments, each conducted in triplicate. Comparisons were made using Student’s *t*-test, with significance levels defined as * *p* < 0.05; ** *p* < 0.01; and *** *p* < 0.001. (Microsoft Excel, version 2408, Microsoft Inc., Redmond, WA, USA).

## 3. Results

### 3.1. Minimum Inhibitory Concentration (MIC) and Minimum Bactericidal Concentration (MBC) for MTZ and CHX Alone or in Combination against P. gingivalis

MTZ’s MIC_50_ was 0.03 mg/mL, while CHX’s MIC_50_ ranged from 0.12 to 0.06 mg/mL (Table 1). In addition, the results showed that for CHX, the MBC (0.12 mg/mL) was equal to or slightly higher than the MIC, whereas, for MTZ, the MBC (0.12 mg/mL) was four times higher than the MIC. Of note, when combined with 0.06 mg/mL CHX, the MIC value of MTZ was one dilution lower than MTZ alone (0.015 mg/mL vs. 0.03 mg/mL, respectively).

### 3.2. Synergistic Effect of MTZ and CHX against P. gingivalis Growth

**Figure 1:**
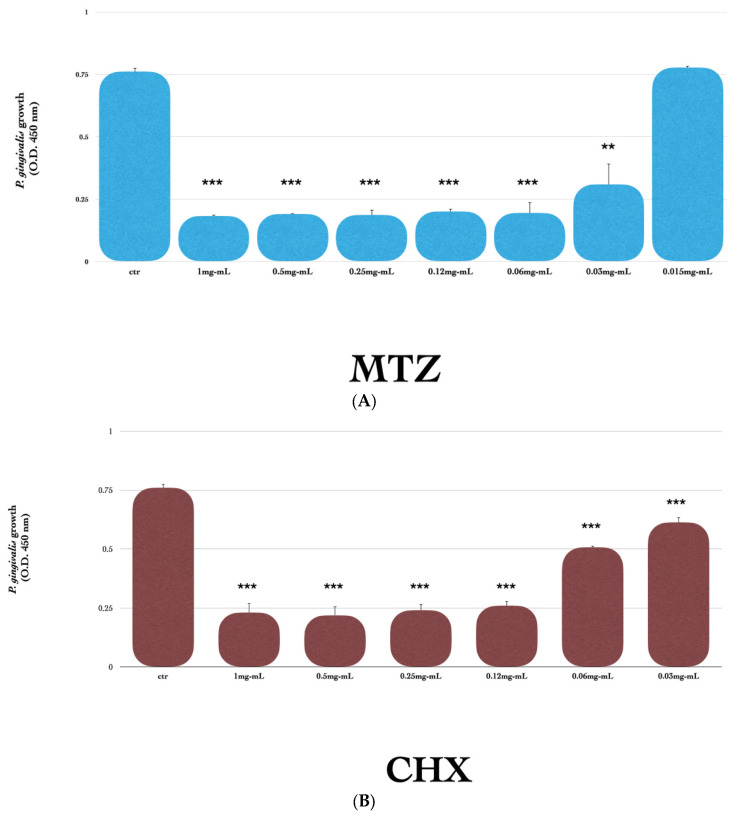

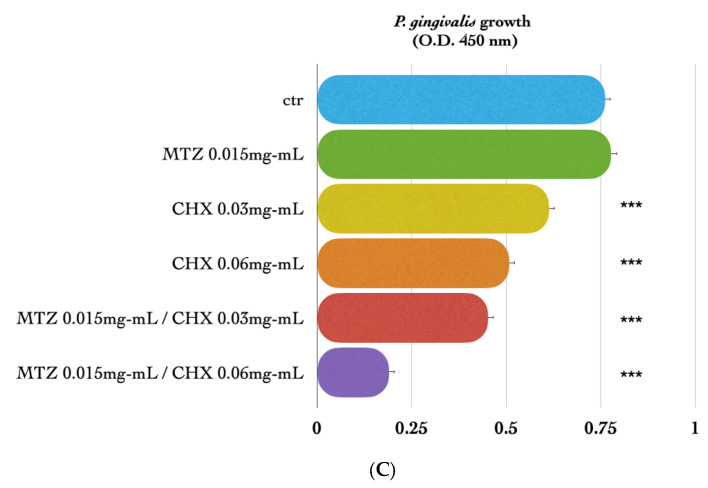
(**A**–**C**) Effect of MTZ and CHX alone or in combination against *P. gingivalis* growth. Control samples were grown in thioglycollate broth without any supplementation (ctr), while compounds were tested alone at different concentrations, ranging from 1 mg/mL to 0.015 mg/mL for MTZ (**A**), from 1 mg/mL to 0.03 mg/mL for CHX (**B**), or in combination with 0.016 mg/mL MTZ plus 0.03–0.06 mg/mL CHX (**C**). Each absorbance value (OD 450) is mean ± SD of three replicates. One representative of three independent experiments is shown. Means were compared by using Student’s *t*-test. Significance level for *p* values was considered as ** *p* < 0.01; and *** *p* < 0.001.

**Figure 2:**
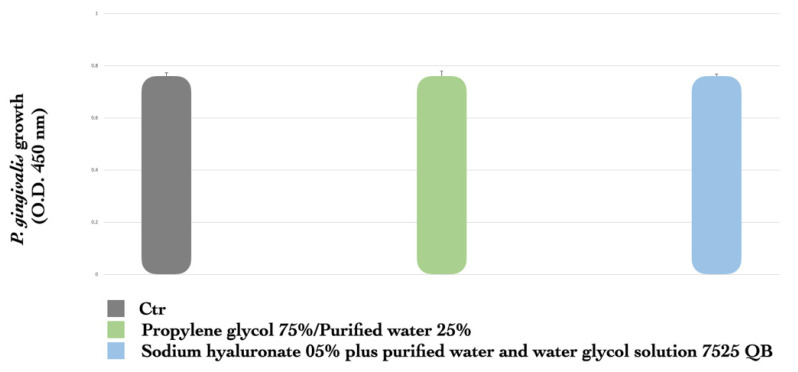
Effect of MTZ or CHX vehicle control on *P. gingivalis* growth. Vehicles propylene glycol 75%/purified water 25%, sodium hyaluronate 0.5% plus purified water, and water glycol solution 7525QB were tested as negative controls for MTZ and CHX. Each absorbance value (OD 450) is mean ± SD of three replicates. One representative of three independent experiments is shown.

The results reported in Figure 1A,B show that both substances exerted a significant inhibitory activity on *P. gingivalis* growth in a dose-dependent manner. Interestingly, as shown in Figure 1C, MTZ at a low and ineffective concentration of 0.015 mg/mL, together with a suboptimal concentration of CHX (0.03 mg/mL), exhibited a significant synergistic inhibitory effect on bacterial growth (50% inhibition vs. control) (*p* < 0.001), and the effect was more remarkable with 0.06 mg/mL CHX (75% inhibition vs. control). The vehicles for both MTZ and CHX, including propylene glycol 75%/purified water 25%, sodium hyaluronate 0.5% plus purified water, and water glycol solution 7525QB, used as the negative controls, did not show any effect against *P. gingivalis* (Figure 2).

## 4. Discussion

The treatment of patients affected by periodontitis is considered challenging for both patients and clinicians. Various approaches, including systemic and local treatments, have been used to treat periodontitis effectively [13,18,19]. It is widely accepted that maintaining an effective drug concentration in the periodontal pockets for a sufficient time is the key to the successful treatment of periodontitis. Therefore, for almost 30 years, antibiotics and antiseptics have been used in the form of direct subgingival administration [20,21,22]. The advantage of this treatment is that the drug concentration after application significantly surpasses the MIC and remains effective for several weeks. This method helps to avoid many side effects associated with systemic antibiotic therapies. Several studies have reported that local antimicrobial therapy is an effective adjunct for treating deep pockets in localized periodontitis [23].

In particular, the local application of CHX through an LDD, such as PerioChip (Dexcel Pharma, Or Akiva, Israel) or PerioCol^®^-CG (Eucare Pharmaceuticals Ltd., Chennai, India), has proven to have beneficial effects in the treatment of periodontitis, showing a significant reduction in the periodontal pocket depth (PPD) score. The highest concentration of the drug in the gingival fluid was maintained for approximately 72 h, ranging from 1400 to 1900 μg/mL. This is significantly higher than the 125 μg/mL concentration required to eliminate 99% of bacteria [24].

Another form of CHX used for intra-pocket applications in the treatment of periodontitis is a plastic gel containing 1.5% CHX. This gel includes 0.5% chlorhexidine digluconate and 1% chlorhexidine dihydrochloride [25].

A few reports have demonstrated a statistically significant improvement in terms of the PPD reduction after using CHX chips in comparison with traditional treatments [26]. In particular, in a study by Pattnaik et al. [27], after a 3-month observation, the PDD score was reduced by 2.36 mm ± 0.84, and the clinical attachment level (CAL) was improved by 2.29 mm ± 0.5 (*p* < 0.001). In another study by Lecic et al., after 3 months of observation, the periodontal pockets were 3.41 mm shallower, and the CAL improved by 1 mm (*p* < 0.001) [28].

Clinically, besides CHX, metronidazole, a nitroimidazole antibiotic, has proven to be effective for treating gingivitis and periodontal disease. Recent studies have suggested that the therapeutic efficacy of MTZ is due not only to its efficacy against periodontal anaerobic pathogens, including *P. gingivalis*, but also to the ability of the drug to improve the killing activity of polymorphonuclear cells (PMNs) against periodontal bacteria [29].

In periodontology, MTZ is utilized as Elyzol Dentagel (A. L. Pharma, Englewood, CO, USA), which contains a 25% concentration of MTZ. This corresponds to 1 g of MTZ benzoate encapsulated in a glycerol matrix and sesame oil [30]. MTZ concentrations of around 120 μg/mL can be detected for at least 8 h, with levels above 1 μg/mL still present at 36 h. When applied to the periodontal pocket twice a week, MTZ solidifies upon contact with the gum fluid, forming crystals that prevent the gel from spilling out. This mechanism helps maintain a concentration above the MIC in the periodontal pockets for an extended period [31].

Despite the efficacy of MTZ against periodontal anaerobic bacteria, this drug alone has a limited effect against facultative bacteria, in comparison with strict anaerobes, such as *Aggregatibacter actinomycetemcomitans* (reclassified *Actinobacillus actinomycetemcomitans*), which is considered the main causative agent of aggressive periodontitis [32]. However, the combination of MTZ with other antibiotics could be a promising therapeutic strategy for the management of periodontal disease. Pandit et al. reported that combined treatment with Minocycline microspheres and MTZ gel improved the clinical parameters, e.g., the PPD and CAL, in patients with periodontitis compared to SRP alone [33].

Pradeep et al. evaluated, for the first time, the clinical and microbiological effects of CHX and MTZ gels applied over a period of 24 weeks, either alone or in combination, in subjects with gingivitis, and found that the beneficial effects, in terms of the reduction in the plaque index (PI) and microbiological count, were significantly higher in the group treated with the CHX-MTZ combination gel compared to those treated with CHX or MTZ gel alone [34].

Moreover, recent in vitro studies have shown that combining silver nanoparticles (AgNPs) coated with CHX or MTZ produces excellent antimicrobial effects against various Gram-positive and Gram-negative bacterial strains (*S. maltophilia*, *S. mutans*, *S. epidermidis*, and *A. lwoffii*), as well as *C. albicans*, suggesting their potential use in treating periodontitis [35].

The main limitations of the present study are intrinsic to its in vitro design and the use of only one bacterium as the target of the investigated concentrations.

However, to the best of authors’ knowledge, this is the first in vitro study investigating the potential combined effect of CHX and MTZ against *P. gingivalis*, which is considered one of the key-stone pathogens of periodontal disease.

The results of the present study show that a low and non-effective concentration of MTZ (0.015 mg/mL) combined with a suboptimal concentration of CHX (0.03 mg/mL) significantly inhibited the in vitro growth of *P. gingivalis* (50% inhibition) in comparison to the untreated control, suggesting an important synergism between the two drugs. Intriguingly, 0.015 mg/mL MTZ combined with 0.06 mg/mL CHX was able to exert bactericidal activity against *P. gingivalis*, as demonstrated by the absence of bacterial growth on the agar plates after 24 h of treatment. These results suggest a promising synergistic interaction between MTZ and CHX and the therapeutic possibility of reducing classic antibacterial drug dosages. This synergistic effect between the two drugs may explain the therapeutic efficacy of CHX-MTZ combination gel in patients with periodontitis, as reported by Pradeep et al. [34].

However, the majority of treatments currently used in periodontitis could be considered imperfect due to the form of administration, as well as the mode and timing of drug release. Although local delivery drug systems may allow for the delivery of high concentrations of drugs directly into the periodontal pocket, there are not sufficient data indicating which LDD system is more effective with respect to the others. Thus, further studies are needed to select the appropriate drugs and their delivery method to reach adequate drug concentrations in the periodontal pockets for a successful support of SRP in the future.

## 5. Conclusions

CHX and MTZ showed a significant synergistic effect against *P. gingivalis* growth. A non-effective concentration of MTZ (0.015 mg/mL) combined with a suboptimal concentration of CHX (0.03 mg/mL and 0.06 mg/mL) were related to a 50% growth inhibition and 99.99% death of *P. gingivalis*, respectively. However, these findings should be tested clinically with randomized controlled trials, also investigating which local drug delivery system could be more effective for maintaining a high drug concentration in the periodontal pockets.

## Figures and Tables

**Table 1:** MIC and MBC values of MTZ and CHX alone or in combination against *P. gingivalis*. MIC = minimal inhibitory concentration; MBC = minimal bactericidal concentration. In combination, CHX was used at suboptimal concentration of 0.06 mg/mL.

Substance	MIC50	MBC
	mg/mL	
MTZ	0.03	0.12
CHX	0.06–0.12	0.12
MTZ plus CHX	0.015	0.015

## Data Availability

Data are available upon reasonable request from the corresponding author.
